# Swelling of Positronium Confined in a Small Cavity

**DOI:** 10.1371/journal.pone.0109937

**Published:** 2014-10-14

**Authors:** Giovanni Consolati, Fiorenza Quasso, Davide Trezzi

**Affiliations:** 1 Department of Aerospace Science and Technology, Politecnico di Milano, Milano, Italy; 2 Department of Physics, Università degli Studi di Milano, Milano, Italy; Washington State University, United States of America

## Abstract

The electron density at the positron (contact density) in the ground state positronium (Ps) formed in condensed matter is generally found to be lower than in vacuum. This is usually attributed to microscopic electric fields which polarize Ps, by acting on the two particles of the atom. In this paper we quantitatively investigate an opposite effect. It is due to the confinement of Ps in small cavities existing in the host solid (*e.g*. free volume in polymers), which increases the contact density. Although this phenomenon is greater, the smaller is the size of the cavity, Ps polarization seems to play anyway a predominant role.

## Introduction

The bound electron-positron system, positronium (Ps), formed in condensed matter shares with Ps *in vacuo* various features; the most important ones are the presence in the ground state of two sublevels (triplet: ortho-Ps, *o*-Ps, parallel spins of the electron and positron; singlet: para-Ps, *p*-Ps, antiparallel spins) characterized by different lifetimes and an energy separation between them [1]. However, there are also significant differences, first of all the possibility to annihilate with an external electron in a relative singlet state (‘pickoff’ process), which changes the lifetimes with respect to those in vacuum (142 ns and 0.125 ns for *o*-Ps and for *p*-Ps, respectively); in particular, the *o*-Ps lifetime in condensed matter can be shortened up to a few ns [2]. Another feature generally found for Ps in matter is a different value, with respect to vacuum Ps, of the ‘contact density’, that is, the electron density at the positron, represented by 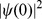, where *ψ* is the Ps wavefunction. Goldanskii [3] introduced for the first time a relative contact density:
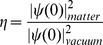
(1)to account for – in a phenomenological way – the perturbation of the positron wavefunction due to the presence of the surrounding matter. A value of the relative contact density less than unity has been attributed to polarization effects on Ps [4], [5] due to local electric fields acting in opposite way on the pair; this effect is expected to be relevant in the presence of polar media [6]. A second factor influencing the Ps contact density is due to the confinement of Ps in cavities, such as defects in solids: in fact, squeezing of the Ps wavefunction should increase the contact density. The smaller the cavity hosting Ps, the stronger the effect should be. Ps can be hosted in a variety of volumes. In amorphous polymers Ps is formed in the free volume holes, which represent the fraction of the total empty space present in the structure, able to accommodate an atomic or molecular probe [7]. In zeolites Ps is located in the cages, while in porous materials (like silica gels) Ps formed in the bulk may diffuse in the structure and eventually be trapped into a pore. In molecular materials the situation is more complex. In some pure crystals (e.g. *p*-terphenyl) Ps is not formed. By doping the host crystal with sufficiently small guest molecules (e.g. anthracene), an enlarged free space is generated in the neighborhood of the guest molecule, which allows Ps formation [8]. In some molecular crystals (such as succinonitrile and adamantane) Ps can be trapped in vacancies [9]. In other molecular crystals (e.g. naphthalene) there is evidence that Ps is not trapped into vacancies, but rather in intermolecular spaces which increase with the temperature [10]. In the present work we used the word ‘cavity’ to mean a generic volume of the solid structure in which Ps can be formed or trapped.

The effective contact density should result from a trade-off between confinement and polarization. Experimentally, it is generally found that the contact density for Ps in matter is lower than in a vacuum, although higher values are not ruled out, in principle [5], [11]. In this work we aim to quantitatively estimate the change of the contact density consequent to the Ps confinement; to this purpose, we will adapt to Ps a theoretical approach already used for hydrogen [12]–[14].

## Theoretical background

We suppose a Ps atom in the ground state and enclosed into a spherical cavity with radius *r*_0_. The potential well is infinite at *r*_0_ and zero for *r*<*r*_0_. Therefore, the wavefunction must vanish at *r*_0_ instead of at infinity, as it is required for vacuum Ps. This boundary condition influences only the radial part *R*(*r*) of the wavefunction, whose equation is generally written in terms of the variable *u*(*r*) = *rR*(*r*) [15]:
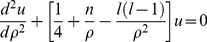
(2)where: 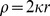; 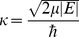; 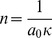; 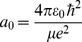; *l* is the azimuthal quantum number and *μ* is the Ps reduced mass. By looking for a solution of the form:
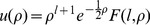
(3)the following equation is obtained:
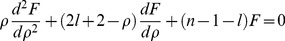
(4)for the confluent hypergeometric function 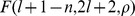 [16].

By expressing *F(l,ρ)* as a power series: 
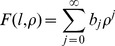
(5)and inserting it into eq. (4) the recursion formula between the coefficients *b_j_* is found:
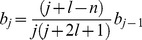
(6)


As it is well known, when the node of the wavefunction is at *r*_0_ = ∞ the series expansion for *F* must break off: the number *n* must be integer (principal quantum number). In the present case this condition is not required and real values of *n* can be expected, with a corresponding shift of the energy levels [14], [17]. In the above formulas *l* = 0, as we will treat only ground state Ps.

## Results and Discussion

**Figure 1:**
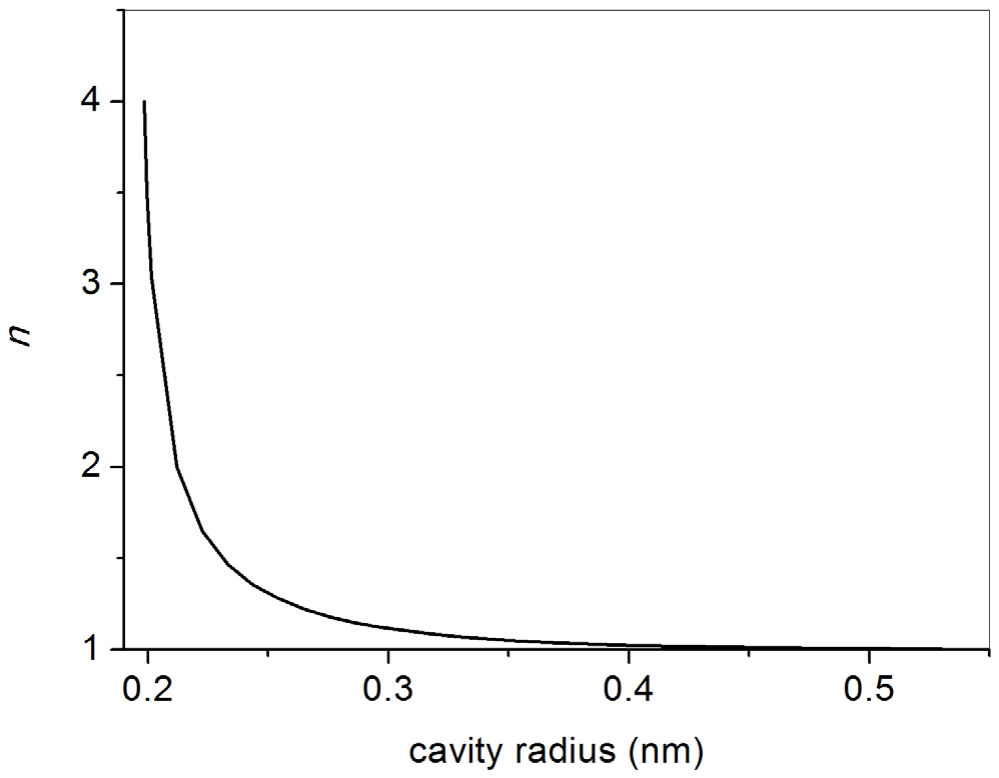
Dependence of the number *n* on the radius *r*_0_ of the cavity trapping Ps.

For *n* real it is necessary to find a relation between *n* and *r*_0_ which makes *F(ρ)* vanish at *r* = *r*_0_; this assures that Ps wavefunction is zero at *r* = *r*_0_. This can be obtained numerically; in fact, a few dozens of terms in eq. 5 are sufficient to discriminate among values of *F* lower than 10^−6^. By requiring that *F* = 0 (or, more precisely, *F*<*ε*, with *ε* ≅10^−6^) for several values of *r*_0_ we get the corresponding values of *n*. A plot of *n* versus *r*_0_ is shown in figure 1; it appears that *n* differs by at least 10% from unity only for *r*_0_<0.30 nm. Clearly, for large values of *r*_0_, *n* tends to its asymptotic limit, equal to 1. Knowledge of the numerical relationship between *n* and *r*_0_ is a prerequisite for the evaluation of the integral which appears in eq. 7 below. There is a limitation to the possible values of *r*_0_, since by decreasing the radius of the cavity hosting Ps, the zero-point energy *E*_0_ of this last increases: *E*_0_ = *h* ^2^/(8*m*_Ps_ *r*_0_^2^). When *E*_0_ equals the Ps binding energy (amounting to – 6.8 eV in vacuum) Ps cannot be formed. Therefore, it is necessary that *r*_0_>0.166 nm, according to the adopted model.

In order to determine the relative contact density it is necessary to find the radial Ps wavefunction at the origin. Indeed, *ψ*(*r*)* = R*(*r*)*Y*_0,0_, where *Y*_0,0_ is the spherical harmonic corresponding to *l* = 0; this is obviously valid for the ground Ps state, the only one of interest in the present work. It follows that 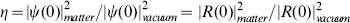. Now *R*(*r*)* = u*(*r*)*/r* and, by using eq. (3), we obtain: 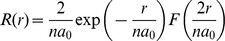 or, by expressing the radial coordinate *r* in the reduced unit *r′* = *r*/*a*_0_: 
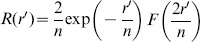
.

Function *F(l,ρ)* in eq. (5) is defined up to an arbitrary constant *b*_0_, which can be fixed by requiring that the integral of the radial part of the wavefunction:
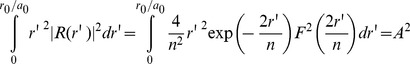
(7)(where *A* depends on both *n* and *r*_0_) is normalized. This condition is satisfied when all the coefficients appearing in eq. (5) are multiplied by *A* ^−1^; then *F*(0) = *b*_0_ = 1/*A* and 
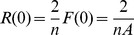
. We note that *F*(0), and whence *R*(0) has a different value for any *r*_0_, as a consequence of the dependence of *A* on *r*_0_.

**Figure 2:**
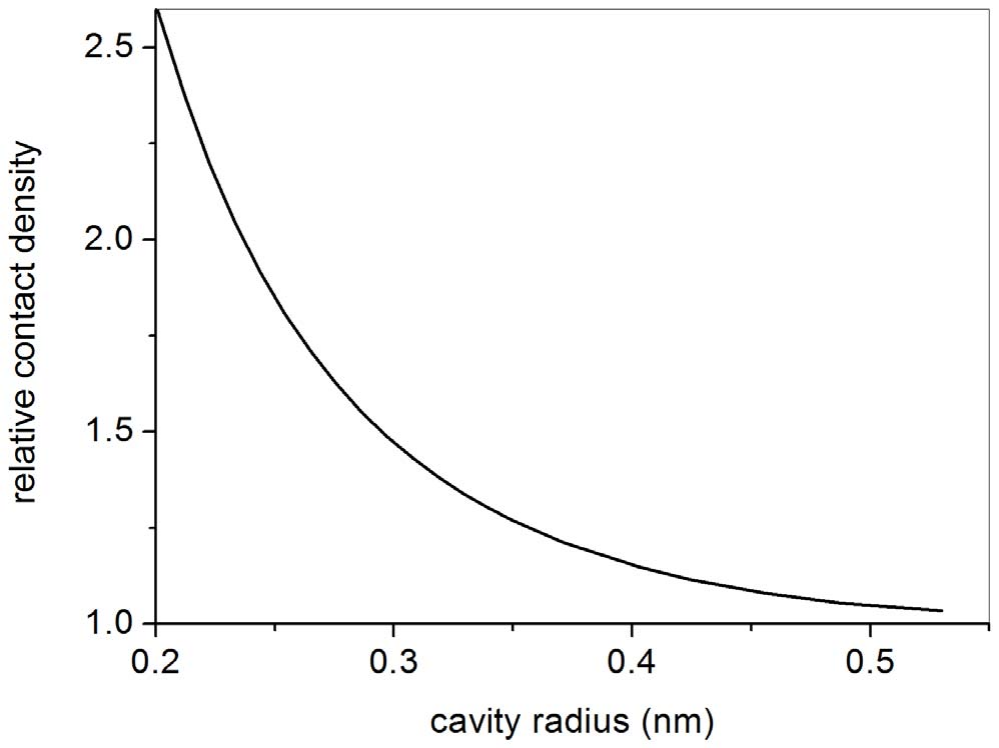
Relative contact density versus the cavity radius *r*_0_; only confinement effects are considered.

Since 
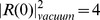
 (in reduced units *r′* = *r*/*a*_0_), the relative contact density can be expressed as follows:



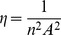
(8)Calculation of the constant *A* appearing in eq. (7) has been carried out numerically for several values of *r*_0_ – and consequently of *n*, according to figure 1. The resulting relative contact density is displayed in figure 2 versus *r*_0_ and shows that *η* increases by decreasing the cavity radius *r*_0_, when only confinement effects are taken into account. We point out that the present discussion is based on the assumption of spherical cavities. Another geometry would imply a different quantitative relationship between *r*_0_ and *n*. But the general trend of the relative contact density as displayed in figure 2 is not expected to change. Indeed, squeezing of the Ps wavefunction due to confinement increases its value at the origin, whatever is the adopted geometry for the cavity, if the normalization of the wavefunction is required.

Experimentally, the relative contact density in media where Ps formation is allowed can be obtained by various methods, the most used being the ‘magnetic quenching’: in the presence of a static magnetic field the Ps sublevels with magnetic quantum number *m* = 0 are mixed and the lifetime of the corresponding triplet sublevel is quenched [18]. It is then possible to determine the relative contact density by means of a fitting procedure on the time annihilation lifetime spectra collected at different applied fields by extracting a ‘quenching ratio’ [19] or by looking for the dependence of the perturbed *o*-Ps lifetime on the magnetic field [20]. The three quantum yield was used, too, to obtain an estimation of the relative contact density [21]; this method turned out useful in the presence of ‘anomalous’ magnetic quenching effects [22]. Comparison between the different procedures showed good agreement, within the experimental uncertainties [21].

Unfortunately, any adopted experimental method cannot split the confinement and the polarization effects on the relative contact density. Nevertheless, it is instructive to recall the experimental results obtained. They confirm that the relative contact density is almost always found to be lower than unity, as already noted in the introduction. The only cases, at the best of our knowledge, where values significantly higher than unity were measured are those of naphthalene [23] and quartz [24]. However, such results were not confirmed [25]–[27]. On the basis of the previous discussion we conclude that Ps polarization overcomes the effect of confinement; the last one could be anyway significant only in small cavities (Figure 2).

**Figure 3:**
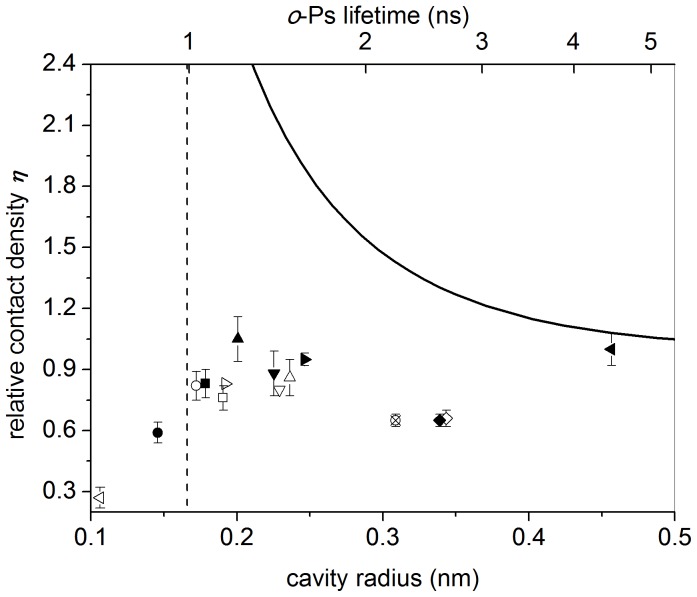
Relative contact density and *o*-Ps lifetime as measured in some molecular solids and polymers (symbols are explained in **Table 1**).

Table 1 and figure 3 display experimental data as obtained in some polymers and molecular solids. These last materials are often characterized by short *o*-Ps lifetimes [28], of the order of 1 ns or even less; therefore, confinement effects should play a significant role. The relative contact densities found in polymers should be considered as rough estimates. Indeed, the presence of a distribution of the free volume holes sizes makes hard to extract a distribution of relative contact densities.

**Table 1:** Relative contact density *η* and *o*-Ps lifetime *τ* in some molecular solids and polymers.

*Material*	*τ* (ns)	*η*	*Symbol in * *fig.3*	*Ref*
**PTFE** a	4.47±0.09	1.00±0.08	◂	[35]
**PPA** b	2.66±0.05	0.66±0.04	◊	[22]
**PE** c	2.60±0.05	0.60±0.06	♦	[35]
**PMMA** d	2.23±0.04	0.65±0.03	⊗	[22]
**a-SiO_2_**	1.59±0.02	0.95±0.03	▸	[27]
**Octadecane**	1.50±0.02	0.86±0.09	Δ	[36]
**p-terphenyl (doped with anthracene)**	1.44	0.80	▿	[37]
**Butyl-PBD** e	1.41±0.07	0.88±0.11	▾	[38]
**PPD** f	1.22±0.02	1.05±0.11	▴	[38]
**p-terphenyl (doped with chrysene)**	1.16	0.83	▹	[37]
**Byphenil**	1.15±0.02	0.76±0.06	□	[26]
**PPO** g	1.07±0.03	0.78±0.04	▪	[38]
**Naftalene**	1.03±0.01	0.82±0.07	○	[26]
**Acenaphtene**	0.88±0.01	0.59±0.05	•	[39]
**Thorium phosphate**	0.70	0.32–0.22	◃	[37]

a Polytetrafluoroethylene.

b Atactic polypropylene.

c Polyethylene.

d Poly(methyl methacrylate).

e 2(4-tert-Butylphenyl)-5-(4-byphenylyl)1,3,4-oxadiazol.

f 2,5-Diphenyl-1,3,4-oxadiazol.

g 2,5-Diphenyl-oxazol.

In figure 3 the relative contact densities are reported versus both the *o*-Ps lifetime (upper *x*-axis) and the cavity size (lower *x*-axis), as obtained by using the Tao-Eldrup equation [29]–[31]: 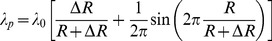
(9)This equation is based on the assumption of a spherical cavity - like the model used in the present work - with effective radius *R*. For convenience of calculations the depth is assumed as infinite, but the radius is increased to 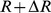, 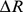 being an empirical parameter which describes the penetration of Ps wave function into the bulk. The electron density is supposed to be zero inside the cavity and constant from *R* to 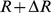. In eq. (9) *λ*_0_ = 2 ns^−1^ is the spin-averaged annihilation rate of *p*-Ps (8 ns^−1^) and *o*-Ps (1/142 ns^−1^) in vacuum, *λ_p_* is the pickoff decay rate. The measured *o*-Ps lifetime *τ*_3_ is the reciprocal of the total decay rate *λ*_3_, sum of the pickoff decay rate and the intrinsic decay rate *λ_i_* (1/142 ns^−1^):
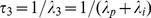
(10)The *λ_i_* contribution, included in Eq. (10) for the sake of completeness, is negligible when *o*-Ps lifetimes are of the order of a few ns. The vertical dashed line drawn in figure 3 represents the minimum value of the cavity radius (0.166 nm) compatible with the Ps zero point energy limitation, as discussed above and dependent on the adopted cavity model.

A reduction of the relative contact density *η* with respect to unity, corresponding to a swelling of Ps atom, is observed in figure 3, on the average. Therefore, polarization is the main effect on Ps contact density, even when the atom is confined in small cavities. To point our this last conclusion we reported in figure 3 also the behaviour of *η* due to bare confinement, already shown in figure 2. Qualitatively, polarization effects can be explained in terms of Van der Waals interactions between Ps and molecules of the medium. Although Ps does not have an electric dipole moment in its ground state, when the surrounding molecules are polar a Ps polarization is expected, through an induced dipole force (Debye force) between the permanent dipole and the Ps induced dipole. Nevertheless, also in non-polar media instantaneous dipole–induced dipole forces (London dispersion forces) can occur. Such forces are weak, but the effect on Ps can be noticeable when the Ps-molecule distance is very short, as in the case of a small sized cavity; this could justify the predominance of polarization effects with respect to the squeezing of Ps wavefunction due to confinement.

## Conclusions

The change of the Ps contact density with respect to Ps in a vacuum, as generally observed, should be attributed to the opposite effects of Ps confinement and Ps polarization, this last being induced by the surrounding molecules. The last effect is stronger than the confinement occurring when Ps is trapped in a small cavity, which squeezes Ps, by increasing the value of its wavefunction at the origin. The results here obtained are based on a simplified model of the cavity, which is supposed to be spherical. Anyway, the experimental relationship between contact density and *o*-Ps lifetime does not imply any particular assumption on the geometry of the cavity: *o*-Ps lifetime can be considered a (non-linear) probe of the cavity size, since by decreasing this last the lifetime decreases, too, due to the increased pickoff rate. Generally, real cages trapping Ps are not spherical; this is not a severe constraint, at least as far as the relationship between *o*-Ps lifetime and cavity size is concerned [32]. Use of a non-spherical geometry [33], [34] would imply a different solution of the Schrödinger equation (*e.g*. in cylindrical coordinates) with respect to the treatment here adopted. In spite of this limitation, the conclusions are not expected to change by using other geometries.
